# Therapeutic potential of APP antisense oligonucleotides for Alzheimer’s disease and down syndrome-related Alzheimer’s disease

**DOI:** 10.1186/s13024-024-00745-5

**Published:** 2024-07-29

**Authors:** Srishruthi Thirumalai, Rickie Patani, Christy Hung

**Affiliations:** 1 UCL Great Ormond Street Institute of Child Health, Zayed Centre for Research into Rare Disease in Children, 20 Guilford Street, London, WC1N 1DZ UK; 2 Human Stem Cells and Neurodegeneration Laboratory, The Francis Crick Institute, 1 Midland Road, London, NW1 1AT UK; 3 Department of Neuromuscular Diseases, Queen Square Institute of Neurology, University College London, London, WC1N 3BG UK; 4 Department of Neuroscience, City University of Hong Kong, Hong Kong, Hong Kong

**Keywords:** Alzheimer’s disease, Down syndrome, Antisense oligonucleotides, Amyloid precursor protein

The amyloid cascade hypothesis of Alzheimer’s disease (AD) suggests that the accumulation of the amyloid-β (Aβ) peptide in the brain is a central event in the disease’s pathology. This hypothesis is strongly supported by both human neuropathological findings and genetic studies. As a result, Aβ-targeted monoclonal antibody (mAb) has been a central focus of efforts to develop drugs aimed at slowing or halting AD progression [1]. Importantly, following the accelerated approval of aducanumab, two further mAbs that target amyloid, lecanemab and donanemab, have received rapid FDA approval. The recent successful clinical trial of lecanemab in symptomatic AD, meeting its primary and secondary endpoints, represents a notable step forward in the battle against this prevalent disease. However, it remains controversial which Aβ species (monomers, oligomers, protofibrils or fibrils) are the most neurotoxic.

Compared to mAb-mediated immunotherapies, antisense oligonucleotides (ASOs) aimed at lowering levels of Aβ either by targeting *APP* mRNA or its enzymes involved in amyloidogenic processing offer an appealing alternative. Previous studies have showcased the potential of ASOs in reducing Aβ species in animal models of AD. For example, OL-1, an ASO targeting the *APP* mRNA region corresponding to the 17–30 amino acid fragment of Aβ [2], reduced APP expression in AD mouse models, including transgenic Tg2576 (APPswe) and SAMP8 mice. Chang et al. developed a splice-switching ASO that induces the skipping of the *APP* exon encoding proteolytic cleavage sites required for Aβ peptide production [3]. Similarly, tau plays a key role in AD pathophysiology [4]. MAPTR_x_ is an ASO designed to reduce tau levels and has shown marked dose-dependent and sustained reductions in the concentration of CSF t-tau in a human phase 1b clinical trial [4].

In the latest issue of *Brain*, Hung et al. further demonstrated the efficiency of APP ASOs in reducing both full-length APP proteins and Aβ-containing aggregates using a human stem cell model [5]. They used a 20-mer (gapmer) APP ASO targeting Exon 5 of the *APP* mRNA and found that nearly all human iPSC-derived cortical neurons contain APP ASOs after 24 hours. Through dose optimization, they showed that APP ASOs are effective in restoring physiological APP levels from what would be expected from three copies back down to the equivalent of would be transcribed from two copies.

Dysfunction of the endolysosomal-autophagy network is emerging as an important pathogenic process in AD [6]. Using super-resolution imaging, Hung et al. showed that APP ASOs rescue endolysosome and autophagy dysfunction in human APP duplication neurons by restoring lysosomal acidity to physiological levels. Accumulation of extracellular Aβ aggregates comprising Aβ peptide oligomers is one of the cellular hallmarks of AD. However, characterization of the aggregates secreted by human iPSC-derived neurons is challenging due to their low concentrations and sub-diffraction limit size. They overcome this technical challenge by using ultrasensitive single-molecule pull-down (SiMPull). Similar to a sandwich enzyme-linked immunosorbent assay (ELISA), SiMPull uses a PEG-passivated surface along with biotin-neutravidin interactions to capture and detect Aβ aggregates using single-molecule fluorescence microscopy. They found that APP ASOs are effective in reducing both intracellular and extracellular (secreted) Aβ-containing soluble aggregates in human cortical neurons.

In summary, their results highlight the potential of APP ASOs as a therapeutic approach for forms of AD caused by duplication of the APP gene, including monogenic AD and Down syndrome-related AD. However, it also raises many questions that warrant further study (Fig. 1). Firstly, while the genetics driving early-onset autosomal dominant AD have placed APP processing and Aβ production at the heart of pathogenesis, it is evident that a combination of genetic and environmental risk factors likely causes sporadic AD (sAD). Therefore, it would be important to perform a screen to identify additional ASOs that can robustly reduce APP levels and Aβ production in the more prevalent sAD cases. Moreover, Alnylam Pharmaceuticals has developed ALN-APP, which fuses a synthetic siRNA targeting APP to a proprietary 2’-O-hexadecyl (C16) lipophilic conjugate, for AD and Cerebral Amyloid Angiopathy. Unlike APP ASO, which targets Exon 5 of *APP* mRNAs by Watson-Crick base pairing and directs their catalytic degradation by RNase H [5], ALN-APP uses an endogenous mechanism whereby siRNAs direct the RNA-induced silencing complex (RISC) to achieve gene knockdown. A direct comparison of the specificity and efficiency of APP ASOs and ALN-APP would provide valuable insights for the field. Secondly, accumulating evidence suggests that Aβ and tau pathologies have synergistic effects [7]. It would therefore be interesting to explore whether APP ASOs can modulate tau protein levels, phosphorylation status, and tau aggregation in human APP duplication cortical neurons. Additionally, it is now increasingly clear that the role of non-neuronal cell populations, particularly glial cells, in AD initiation and progression is more substantial than previously recognized [1, 8]. Many genetic risk factors linked to late-onset AD prominently involve glial cell populations, particularly astrocytes and microglia. These risk genes are functionally related to processes such as endocytosis and lysosomal activity [9]. Therefore, it would be interesting to investigate whether human astrocytes and microglia uptake APP ASOs as efficiently as human cortical neurons. Additionally, would combining ASO and mAb immunotherapies be more effective than monotherapy due to the disease’s complexity?

**Fig. 1:**
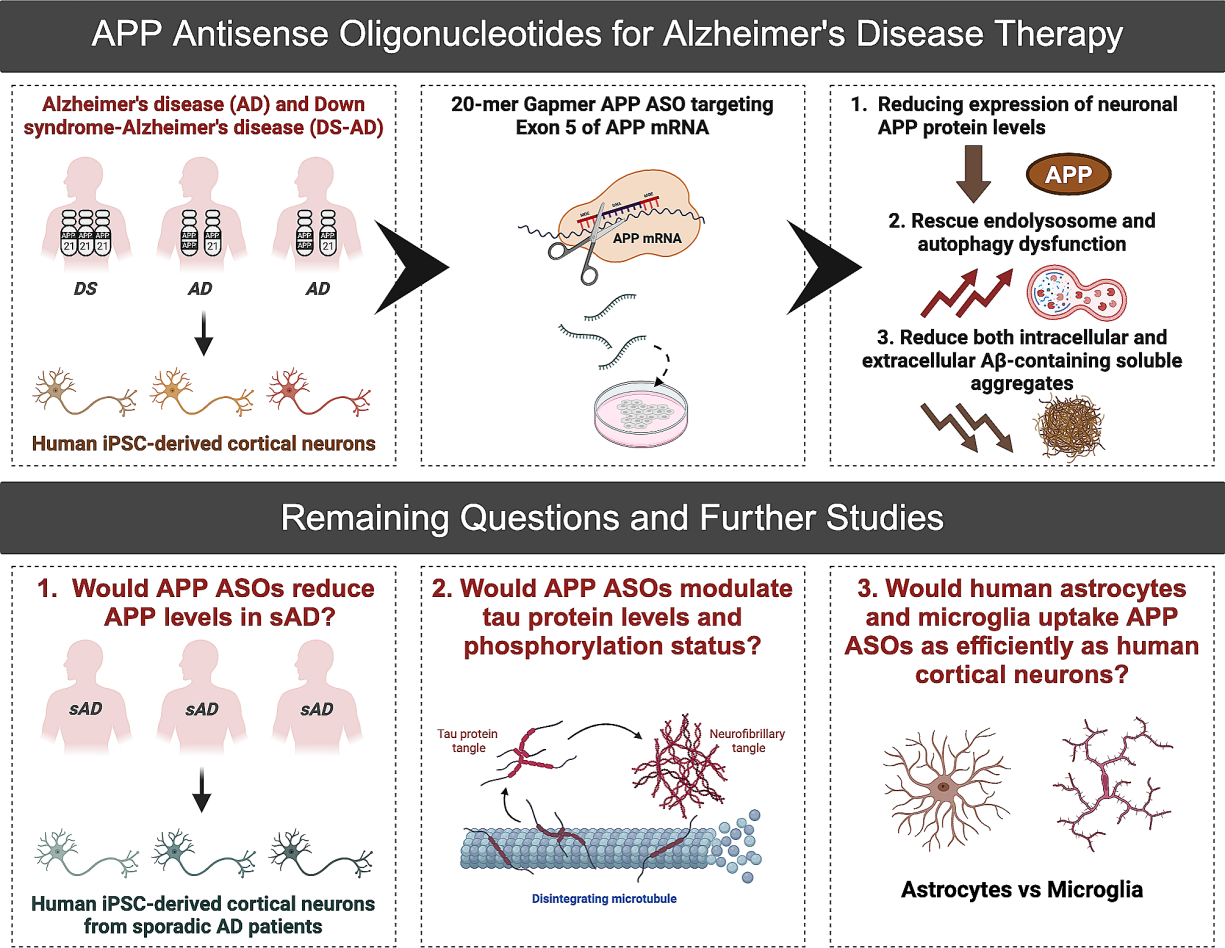
Therapeutic potential of APP antisense oligonucleotides for Alzheimer’s disease and Down syndrome-related Alzheimer’s disease. Antisense oligonucleotides targeting APP are an effective approach to reduce APP protein levels and rescue endolysosome and autophagy dysfunction in APP duplication human induced pluripotent stem cell-derived cortical neurons. Importantly, using ultrasensitive single-aggregate imaging techniques, APP targeting ASOs significantly reduce both intracellular and extracellular Aβ-containing aggregates. This exciting study highlights the potential of APP ASOs as a therapeutic approach for forms of AD caused by duplication of the APP gene, including monogenic AD and AD related to Down syndrome

Additional factors, such as off-target effects, delivery challenges, and optimal timing for intervention, must also be considered for successful therapeutic application of ASOs [10]. While safe and localized delivery to the brain can be achieved through intrathecal administration, it remains an invasive procedure [10]. The roadmap for ASOs being clinically impactful has been realised for several neurological disorders including spinal muscular atrophy (using a splice switching MIXmer ASO to promote exon 7 inclusion in *SMN2* and thereby increase functional SMN expression) and SOD1-related amyotrophic lateral sclerosis (using a GAPmer ASO to reduce SOD1 levels). Since ASOs dosed into cerebrospinal fluid distribute broadly throughout the central nervous system, understanding the differences in activity and duration of action of APP ASOs between different CNS cell types would be essential for further enhancements in design and ultimate potency of APP ASOs for AD therapy in the future.

## Abbreviations

AD: Alzheimer’s disease
Aβ: amyloid-β
ASOs: antisense oligonucleotides
ELISA: enzyme-linked immunosorbent assay
sAD: sporadic Alzheimer’s disease
SiMPull: single-molecule pull-down
iPSC: induced pluripotent stem cell
mAb: monoclonal antibody
